# Clade 2.3.4.4b H5N1 neuraminidase has a long stalk, which is in contrast to most highly pathogenic H5N1 viruses circulating between 2002 and 2020

**DOI:** 10.1128/mbio.03989-24

**Published:** 2025-02-26

**Authors:** Enikő Hermann, Florian Krammer

**Affiliations:** 1 Ignaz Semmelweis Institute, Interuniversity Institute for Infection Research, Medical University of Vienna, Vienna, Austria; 2 Department of Microbiology, Icahn School of Medicine at Mount Sinai, New York, New York, USA; 3 Center for Vaccine Research and Pandemic Preparedness (C-VaRPP), Icahn School of Medicine at Mount Sinai, New York, New York, USA; 4 Department of Pathology, Molecular and Cell-Based Medicine, Icahn School of Medicine at Mount Sinai, New York, New York, USA; University of Hong Kong, Pokfulam, Hong Kong

**Keywords:** H5N1, neuraminidase, influenza, neuraminidase stalk

## Abstract

Since 2020, H5N1 highly pathogenic avian influenza (HPAI) viruses of clade 2.3.4.4b have been rapidly spreading in wild birds but have also caused a large number of mammalian infections and more than 70 known human cases. Importantly, this H5N1 clade has also crossed the species barrier into dairy cattle in the US in late 2023/early 2024. The neuraminidase (NA) protein of the N1 subtype can feature truncations in its stalk domain, which have been identified as putative virulence factors in poultry but seem to have a negative impact on transmission in mammals. Since its emergence, the vast majority of HPAI H5N1 A/goose/Guangdong/1/1996-lineage isolates have featured this truncated version of the NA stalk domain. Here, we report that this changed with the 2020 expansion of clade 2.3.4.4b H5N1 and that the majority of isolates—including the strains circulating in dairy cattle—feature a long NA stalk domain.

**IMPORTANCE:**

While the truncated version of the N1 neuraminidase stalk domain may be associated with increased virulence in poultry, the long version of the stalk domain has been associated with increased transmissibility in mammals. The vast majority of highly pathogenic H5N1 of clade 2.3.4.4b that is currently circulating globally features the long stalk version of the neuraminidase, which may increase the risk for these viruses to become human-to-human transmissible.

## OBSERVATION

In 2020, clade 2.3.4.4b of the H5N1 A/goose/Guangdong/1/1996 highly pathogenic avian influenza (HPAI) virus lineage started to rapidly expand in Eurasia. In late 2021/early 2022, the virus crossed the Atlantic from Europe via Iceland in migratory birds followed by additional introductions from Asia (1, 2). This clade then spread rapidly in the Americas and even reached Antarctica. Clade 2.3.4.4b HPAI H5N1 is now well established in global wild bird populations (except for Australia), has caused widespread issues in domestic poultry, has caused widespread (dead-end) infections in scavenging mammals, is suspected to have caused outbreaks with sustained mammal-to-mammal transmission in fur farms and marine mammals, and has caused a sustained outbreak in dairy cattle (3, 4) in the United States. Furthermore, at least 70 human cases have been reported, with six severe cases with two mortalities (one in the United States and one in China), albeit most have been described as mild (5–7).

After its emergence in the 2010s in Asia, clade 2.3.4.4 H5 has extensively reassorted with other avian influenza viruses and has circulated as what is called H5NX viruses, with X being mostly N2, N5, N6, N8, or N9 neuraminidases (NAs) (8). The subclade 2.3.4.4b, which is the cause of the current panzootic outbreak, however, is again an H5N1 virus. In addition to the polybasic cleavage site of the hemagglutinin (HA), which is thought to be the main virulence factor of HPAI, the NA is believed to potentially contribute to pathogenicity as well. NA activity is important for the release of budding virions from infected cells and for the penetration of mucosal fluids. This activity of the NA (the receptor-destroying enzyme) is in balance with the activity of the HA (the receptor-binding protein), and this balance governs fitness in different species. The typically long stalk of the NA (wild type) can be truncated, which can have an impact on NA activity. NA stalk truncations have been found in N1, N2, N3, N5, N6, and N7 subtypes and have been mostly associated with gallinaceous hosts. However, it is mechanistically unclear why truncations are associated with these specific subtypes and not with others. Since the stalk domain often features glycosylation sites, truncations of the stalk domain may also alter the overall glycosylation state of NA, which may modulate virus stability (9), with less glycosylation thought to be associated with lower stability. Truncations of the stalk domain in HPAI H5N1 viruses have been linked to increased pathogenicity in mice (10, 11), chickens (11), and mallard ducks (12) and potentially increased transmissibility in chickens (13). However, the increase in pathogenicity in chicken was not observed in all experimental settings (10). In the context of H7N1, decreased N1 stalk length led to higher pathogenicity in chickens as well but reduced replication and shedding in Pekin ducks (14). An increase in pathogenicity in mice was also observed in pandemic H1N1 and H7N9 viruses that had a shorter NA stalk (15, 16). However, a truncated version of the N1 NA from H5N1 (in a pandemic H1 virus context) did not support droplet transmission in ferrets, showed reduced ability to penetrate human mucus, and led to viral aggregation (17). Increasing the stalk length led to increased transmission in ferrets in a direct contact and respiratory droplet transmission setting, suggesting that H5N1 viruses with a long stalk may have a higher potential to cause mammal-to-mammal transmission compared to the short stalk version (17).

**Fig 1:**
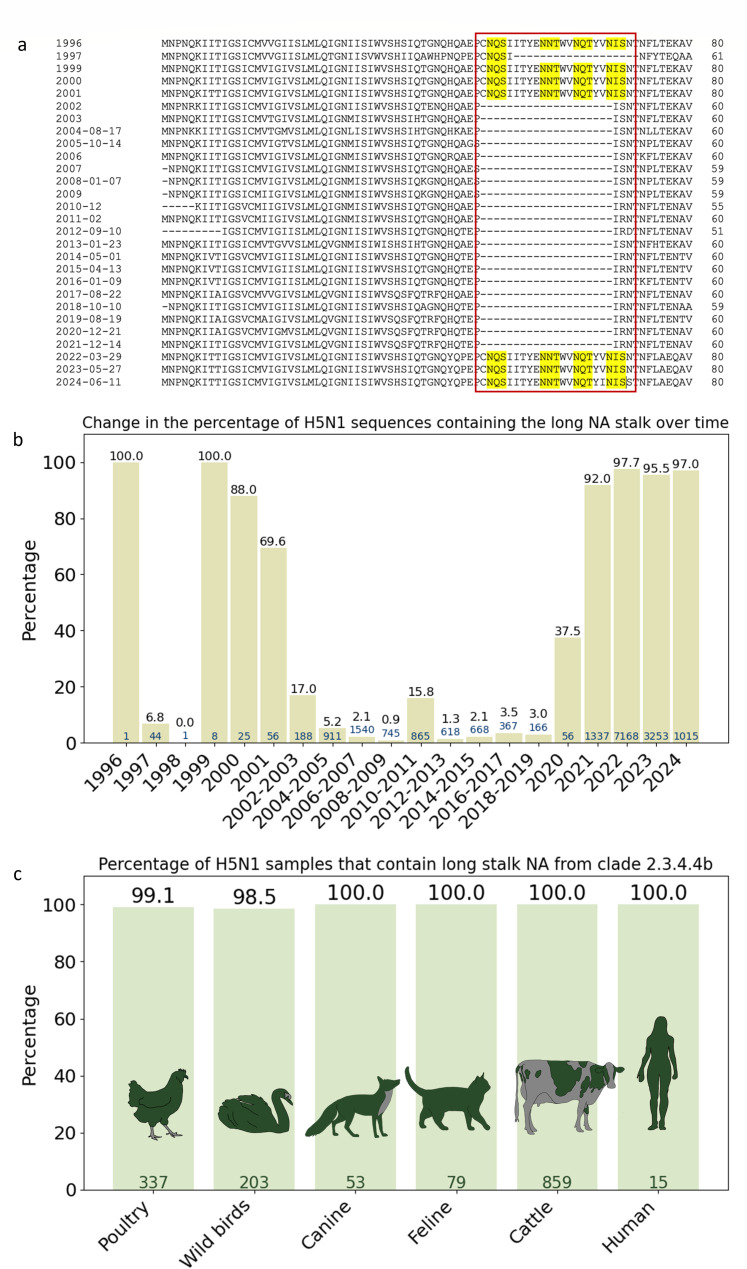
Overview of long and short N1 NA stalk sequences. (a) Multiple sequence alignment of one representative sequence of H5N1 neuraminidase, downloaded from NCBI Virus (18), from every available year from 1997 to 2024. The insertion in the stalk region that appeared first in 1996 and became the dominant “long stalk” variant from 2021 to 2022 is marked with a red rectangle, predicted N-glycosylation sites included in this region are highlighted in yellow. As a reference, the A/goose/Guangdong/1/1996 sequence was used. NCBI accession numbers of the used sequences are as follows, starting from 1997: ACZ45121.1, ACZ45106.1, ACZ45112.1, ACZ45114.1, ACA47584.1, BAM85821.1, AGH30708.1, ABU99043.1, ABV24001.1, AGK72265.1, AGJ73095.1, AUD40562.1, AER09772.1, BAM62219.1, AIZ06790.1, AGM16067.1, APG38584.1, AMX74824.1, ARX75171.1, QHQ71777.1, QEP94697.1, QIC52185.1, UMW88849.1, UTS97654.1, WHA13083.1, WWE95248.1, and XDL05563.1. Alignment was done with Clustal Ω (19). (b) Percentage of NA sequences from H5N1 viruses per year that contain the long stalk N1 (as defined in the "Methods" section in the Supplemental Material). (c) Percentage of NA sequences from H5N1 viruses from clade 2.3.4.4b that contain the above long stalk N1, per host. Numbers at the base of the bars indicate the total number of sequences, and numbers on top indicate the percentage having the long stalk. For avian species, the search was limited to samples collected from 1 January 2024, and for cattle sequences, from 1 January 2023 (downloaded on 15 July 2024 or 19 July 2024). Poultry was defined as any sequences containing the words “chicken,” “turkey,” “domestic duck,” “domestic goose,” “domestic,” or “poultry” in their header. All other avian samples were classified as wild birds.

To compare the sequence evolution of N1 neuraminidase from the A/goose/Guangdong/1/1996-lineage HPAI H5N1 viruses, we downloaded one example sequence from each year from the NCBI Virus server and aligned them. Sequences were from a diversity of species, including chickens, ducks, turkey, or Baikal teal. While the original A/goose/Guangdong/1/1996 lineage had a long NA stalk, we observed a 20 amino acid-long truncation in the stalk region (referred to as “short stalk” from here on) of N1 appearing in the late 1990s. From 2002 onward, the long stalk was then mostly absent until it reappeared in 2022 (Fig. 1a; Fig. S1). It is notable that the long stalk also adds four putative N-glycosylation sites (NQS, NNT, NQT, and NIS).

To investigate the appearance and reappearance of the long stalk NA more accurately, we analyzed all H5N1 sequences available at the Global Initiative on Sharing All Influenza Data (GISAID) EpiFlu server (20), per year. H5N1 sequences with the long stalk N1 represent the majority of sequenced H5N1 samples between 1999 and 2001, but afterward, the fraction with the long stalk gradually declined (Fig. 1b; Fig. S2). However, viruses that express the long N1 stalk kept circulating at low levels, and samples of H5N1 HPAI viruses with long stalk sequences have been identified each year, which is in line with previous findings (17). The long stalk N1 then became dominant again from 2021 onwards in H5N1 samples.

The vast majority of currently circulating clade 2.3.4.4b H5N1 viruses express a long stalk NA (Fig. 1c): 100% of mammalian samples and 99% of avian samples, from a variety of species, including chicken, mute swans, foxes, cats, and dairy cows among others. Interestingly, out of the long stalk sequences, ~65% and ~80% of the H5N1 viruses isolated from wild bird and poultry species, respectively, in 2024 contain a long stalk sequence identical to the one from 1996 (“CNQSIITYENNTWVNQT”), while this percentage is higher for mammalian species (88.2%–100%) (Fig. S3). This potentially suggests faster evolution of the long stalk region in wild birds. After analyzing all “short” sequences identified in clade 2.3.4.4b H5N1 manually (13,335 total sequences, out of which 150 were identified as short), we found that they contain novel deletions that do not match the deletion observed between 2000 and 2020 (Fig. S4). These new deletions were observed mainly in the West African region, with a few isolated examples in Europe and North America between 2021 and 2024. However, they do not seem to be widespread. Altogether, these findings show that clade 2.3.4.4b H5N1 viruses express long stalk N1, and the evolution of stalk composition and length is ongoing.

**Fig 2:**
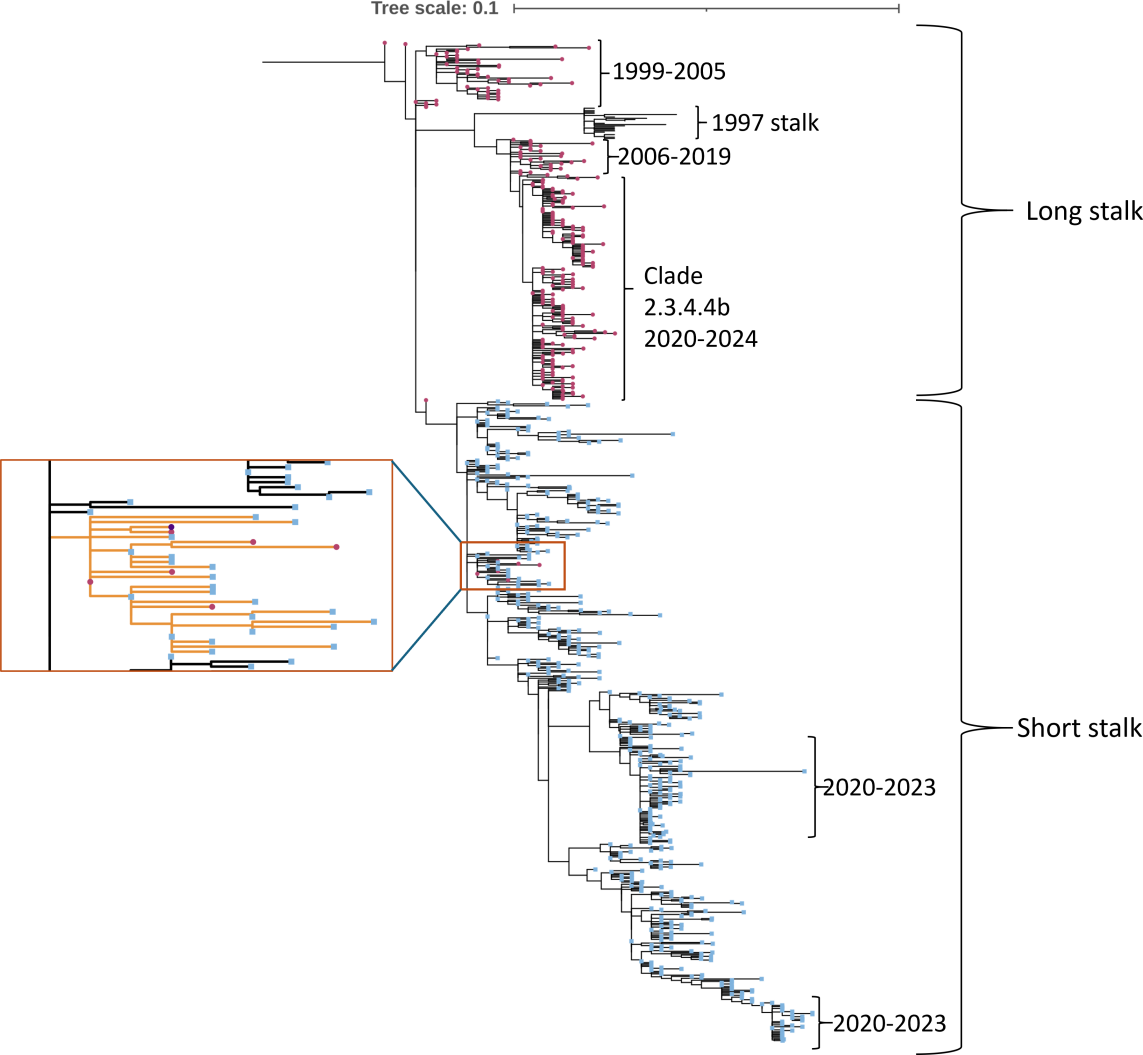
A phylogenic tree created using H5N1 NA sequences with long (blue squares) or short stalk (pink circles). The tree was rerooted to an N4 sequence (A/mallard/Sweden/24/2002, EPI267067 (GISAID)) included in the analysis (not shown on the tree). The orange inset zooms into the only branch (marked with orange) where both long- and short-stalk sequences appear, along with the ancestral A/goose/Guangdong/1/1996 sequence (purple circle). The “1997 stalk” represents a different branch with a distinct deletion compared to what is observed from 2000. The tree was created using the FastTree one click workflow of the Ngphylogeny.fr (21) server and visualized with iTOL (22). The groups marked with years indicate that the branch contains sequences from that time period, with one or two outliers. The tree scale and branch lengths refer to the number of substitutions per site.

Another observation when looking at the head domain of N1 neuraminidases was that some sites in the head domain of N1 that mutated from 1998 to 2019 seemingly reverted these mutations in 2020–2021 to what was observed in 1997 or between 1998 and 2001 (Fig. S5a). In contrast, if we analyze the evolution of these sites only in samples that contain the long stalk N1, we can see that most of them do not evolve throughout the years (Fig. S5b). This suggests that H5N1 viruses with the long stalk NA have been present, mostly as non-dominant variants since at least 1996 (earlier sequence data on H5N1 viruses is lacking)—either continuously or through reassortment with avian HXN1 viruses. This conclusion is supported by a phylogenic tree created using H5N1 NA head domain sequences with long and short stalks (but removing the stalk portion from the phylogenetic analysis) from all years from 1996 to 2024 (Fig. 2). Even only considering the head domain, two distinct groups form, containing long or short stalk domains. Only one branch contains both long- and short-stalk sequences (Fig. 2, orange inset), which also includes the ancestral A/goose/Guangdong/1/1996 sequence. The remaining sequences (with one exception from 2008) in this branch are from the late 1990s or early 2000s, explaining the similarity of head domains. The shortened stalk observed in 1997 is different from the one appearing from 2000 (Fig. 1a) forming a separate group in the phylogenic tree, potentially indicating a separate deletion (or reassortment) event. This variant has not been detected in H5N1 since 2005. It is notable that while the long stalk branches can be clearly separated by observation years, with one or two outliers (Fig. 2), the sequential separation is not clear at all in the case of the short stalk variants. For example, samples from 2020 to 2024 with short stalk are found in two separate groups. All clade 2.3.4.4b sequences included in this tree are long stalk variants, and short stalk variants from this period do not belong to clade 2.3.4.4b. It may be possible that the early spread of H5N1 in Eurasia was more driven by poultry-to-poultry transmission (where the short stalk version may have increased fitness), while current transmission may be driven in large parts by wild birds (where the long stalk version may have a fitness advantage). This may potentially explain the change from short stalk dominance to long stalk dominance.

Interestingly, in clade 2.3.2.1c, which has recently caused severe human cases and deaths in Cambodia (23), almost none of the available N1 sequences have a long stalk (0.2% of the 1,322 on GISAID on 27 August 2024).

Recently, a human H5N1 infection has been described in Missouri for which the source of infection is unknown (24), (A/Missouri/121/2024, EPI_ISL_19413343-EPI3556415 on GISAID). The stalk region of this N1 virus is long and shows an additional mutation, with an additional putative glycosylation site, as it includes an I30T mutation (sequential numbering, Fig. S6). Of note, the D1.1 genotype, which has caused two recent severe infections in humans in North America (with one death [7]), has acquired a different N1 from North American avian influenza viruses through reassortment. However, this NA also has a long stalk domain.

In conclusion, in the currently circulating clade 2.3.4.4b H5N1 viruses, neuraminidase mainly has a long stalk, which could lead to increased potential for mammal-to-mammal transmissions, based on previous work (17).

## SUPPLEMENTAL MATERIAL

Supplemental Material mbio.03989-24-s0001.pdf Supplemental text and figures.

ASM does not own the copyrights to Supplemental Material that may be linked to, or accessed through, an article. The authors have granted ASM a non-exclusive, world-wide license to publish the Supplemental Material files. Please contact the corresponding author directly for reuse.
