# Supplementary material for: Clade 2.3.4.4b H5N1 neuraminidase has a long stalk, which is in contrast to most highly pathogenic H5N1 viruses circulating between 2002 and 2020
Source: mBio. 2025 Feb 26;16(4):e03989-24. doi: 10.1128/mbio.03989-24 (PMC11980542; doi:10.1128/mbio.03989-24)
Supplement: Supplemental Material — Supplemental text and figures. [file mbio.03989-24-s0001.pdf]

**Supplemental Material**

# Clade 2.3.4.4b H5N1 neuraminidase has a long stalk which is in contrast to most highly pathogenic H5N1 viruses circulating between 2002 and 2020

Enikő Hermann[1], Florian Krammer[1,2,3,4,a]

[1] Ignaz Semmelweis Institute, Interuniversity Institute for Infection Research, Medical University of Vienna, Vienna, Austria
[2]Department of Microbiology, Icahn School of Medicine at Mount Sinai, New York, NY, USA
[3]Center for Vaccine Research and Pandemic Preparedness (C-VaRPP), Icahn School of Medicine at Mount Sinai, New York, NY, USA
[4]Department of Pathology, Molecular and Cell Based Medicine, Icahn School of Medicine at Mount Sinai, New York, NY, USA

[a] Corresponding author: florian.krammer@mssm.edu and florian.krammer@meduniwien.ac.at

**Methods**

**Sequence alignment of a single sequence per year:** Using the random sequence download tool of NCBI Virus [1], we downloaded a representative protein sequence from each collection year, specifying the genotype as H5N1 among influenza A viruses, having the protein "neuraminidase" between 1997-2024. The A/goose/Guangdong/1/1996 sequence was included manually. These sequences were aligned using Clustal Ω [2]. The glycosylation sites were predicted with the NetNGlyc 1.0 server [3].

**Identifying sequences with long stalk**: For each marked year or span of years all neuraminidase sequences from H5N1 viruses were downloaded from GISAID. For sequences per host, the genotype 2.3.4.4b was specified, for avian species the search was limited to samples collected from 01/01/2024, and for cattle sequences from 01/01/2023 (downloaded on 15/07/2024 or 19/07/2024). Poultry was defined as any sequences containing the words "chicken", "turkey", "domestic duck", "domestic goose", "domestic", or "poultry" in their header, all other avian samples were classified as wild birds. Sequence sets were filtered to only include ones with over 400 amino acids. All sequences with 469 amino acids were classified as "long stalk". Any further sequences containing the amino acid sequence "CXQSIIXYENNTWVNQT" were identified as "long", which is 3 amino acids shorter than the full "CNQSIITYENNTWVNQTYVN" insertion sequence, and can be any amino acid at the two positions marked with X. We chose this sequence because we observed variations in the sequence logos at the "X" positions, and in the YVN stretch in samples from 1998-2001 and 2021-2024 (Figure S2). Any other sequences were classified as "short stalk". For analyzing clade 2.3.4.4b H5N1 sequences, a complete set was downloaded on September 5$^{th}$ 2024 from GISAID, and the resulting "short stalk" set was analyzed manually.

A set of 2.3.4.4b H5N1 samples with collection dates between January 1$^{st}$ 2024 and August 21$^{st}$ 2024 (the date before the collection of A/Missouri/121/2024) was obtained from GISAID and filtered for complete length sequences containing 469 residues, aligned with Clustal Ω and a consensus sequence was created with EMBOSS Cons. This was compared to the A/Missouri/121/2024 sequence (Figure S6).

**Phylogenic tree:** To create the phylogenic tree the amino acid sequences for each year or set of years that were classified as long or short were collected. To work with full sequences, only sequences from the long- or short stalk sets having 469 or 449 residues were used. To include the stalk variation from 1997 (Figure 1a), all sequences were searched for the sequence "CNQSINF", and ones containing 450 amino acids were used. From the filtered sequences for each set of years up to 50 long- and short stalk sequences were chosen randomly and used to build the tree together with all sequences having the variation from 1997. To define the heads only, sequences having short, long and "1997" stalks were taken from the 91$^{st}$, 71$^{st}$, and 70$^{th}$ amino acid residues respectively. As an outgroup, the sequence of an N4 neuraminidase was included (A/mallard/Sweden/24/2002, EPI267067), where the head was defined from the 91$^{st}$ amino acid. This resulted in a set of N-terminally truncated amino acid sequences that had the same length. The dataset only containing head sequences was further filtered for duplicates. This resulted in 795 sequences. A phylogenic tree was created from these sequences using NGPhylogeny.fr [4], with the FastTree [5], [6] One click workflow. This uses the MAFFT [7] software for multiple sequence alignment, and the BMGE software [8] for curation of the aligned sequences. FastTree calculates approximately-maximum-likelihood trees, and instead of traditional bootstrapping,

local support values, using the Shimodaira-Hasegawa test, with 1000 resamples [9]. The phylogenic tree was drawn using iTOL (Interactive Tree of Life) [10]. The tree was rerooted at the N4 sequence (not shown on Figure 2).

**Evolution of amino acids:** After multiple sequence alignment using Clustal Ω for each year or span of years marked in Fig. S3, a consensus sequence was created using the EMBL-EBI EMBOSS Cons server [11], and the resulting sequences from 1998-2001 vs 2018-2019 were compared for differences. The changes at these positions in the head domain of neuraminidase (amino acids starting from amino acid 90, N2 numbering) were examined further with sequence logos created from alignments with Clustal Ω, including only the sequences containing at least 300 amino acid residues, using Weblogo 3 [12]. The same method was used for sequences that were defined as having a long stalk from each set of years, except in this case all sequences were used that contained the long stalk, independent of their length.

**Graphing:** Plots were created with Matplotlib. Scripting was helped by ChatGPT.

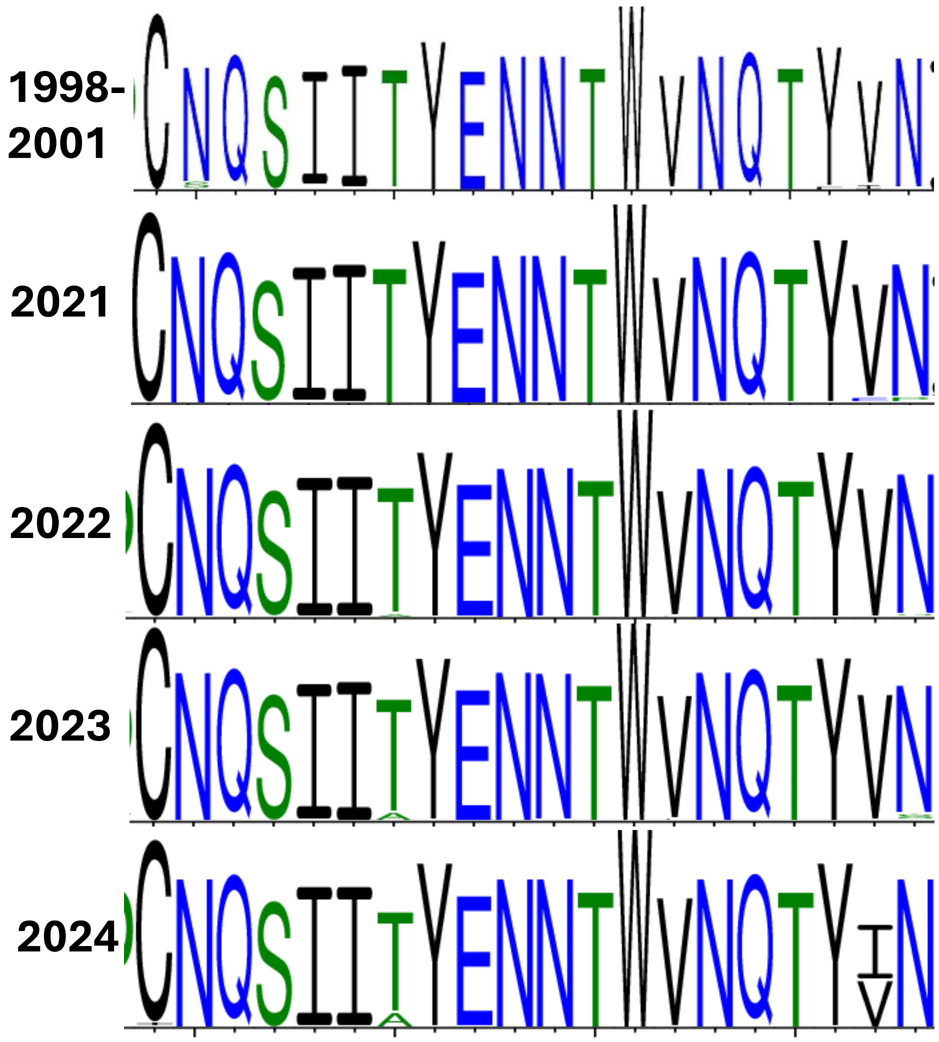


Figure S1: Sequence logo of the long stalk motif, from samples from 1998-2001 and 2021 to 2024. Logos were created with Weblogo 3.

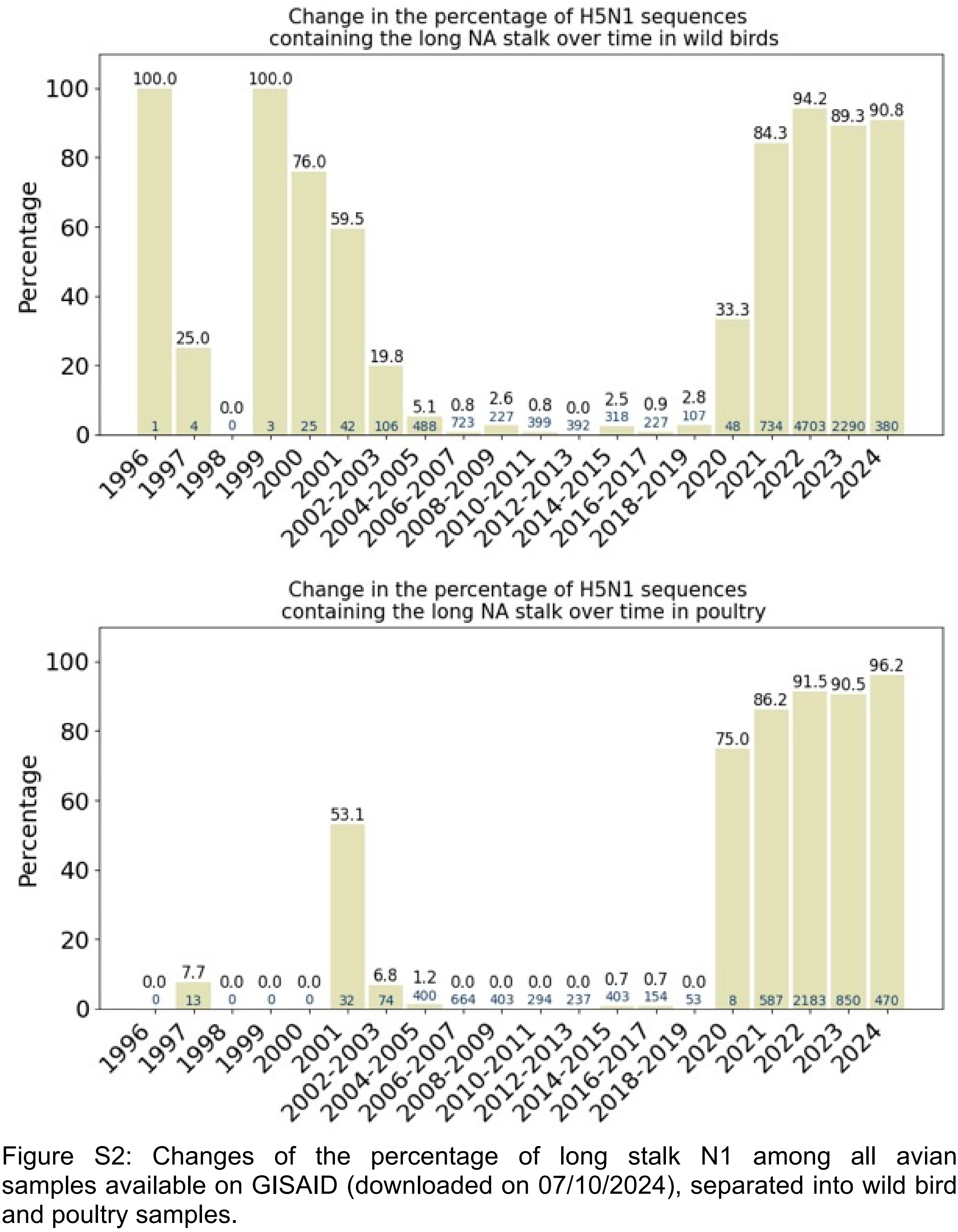


Figure S2: Changes of the percentage of long stalk N1 among all avian samples available on GISAID (downloaded on 07/10/2024), separated into wild bird and poultry samples.

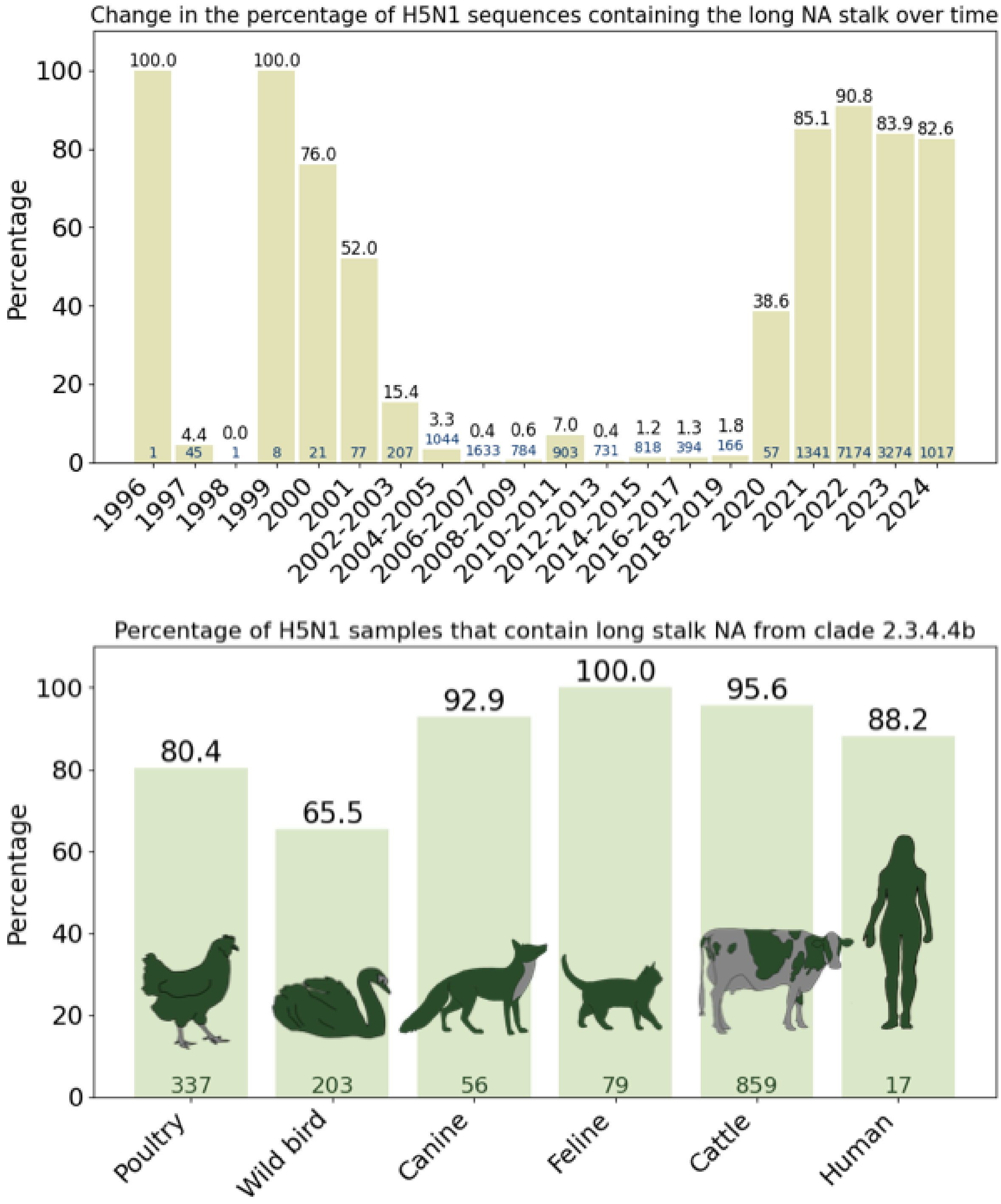


Figure S3: Percentage of long stalk isolates if the search sequence CNQSIITYENNTWVNQT is used, with no variations considered at the C**N**Q or I**T**Y positions. While for mammalian species, the inclusion of the variation is mostly irrelevant, for avian species it is clear that variations appear.

```
A/duck/Burkina_Faso/BKF11_24VIR3019-11/2024_-_EPI_ISL_19171212_-_EPI3343249            MNPNQKIMTIGSICMVIGIVNLMLQIGNIISIWVSHSIQTGNQY----------------------VNISNTNFLAE
A/chicken/Niigata/22E11T/2023_-_EPI_ISL_18286343_-_EPI2751943                           MNPNQRIITTGSICMVIGIVSLMLQIGNIISIWVSHSIQTGNQHQPE---------------------INNTNFLAE
A/chicken/Iowa/23-034847-001/2023_-_EPI_ISL_18749468_-_EPI2906341                       MNPNQKITTIGSICMVIGIVSVMLQIGNIISIWVSHSIQTGNQYQ--------------------------TNFLAE
A/Larus_ridibundus/Spain/CR4063/2023_-_EPI_ISL_18983379_-_EPI3115339                    MSPNQRIITTGSICMVIGIVSLMLQIGNIISIWVSHSIQTGNQYV--N--------------------ISNTNFLAE
A/white-tailed_eagle/Estonia/TA2111864-2_21VIR7512-6/2021_-_EPI_ISL_7778755_-_EPI1945380 MNPNQKITTIGSICMVIGIVSLMLQIGNIISIWVSHSIQTGNQYQPEPCNQSII-----------------------
A/fox/Michigan/22-014536-004/2022_-_EPI_ISL_15078245_-_EPI2182062                       MNPNQKITTIGSICMVIGIVSLMLQIGNIISIWVSHSIQTGNQYQPE---------------------PCNTNFLAE
A/avian/Nigeria/739_22VIR3286-27/2021_-_EPI_ISL_17414596_-_EPI2505988                   MNPNQKIITIGSICMVIGIVSLMLQIGNIISIWVSHSIQTGXQY----------------------VNISN---LAE
A/wild_duck/Shandong/628/2011_-_EPI_ISL_148126_-_EPI475581                              MNPNQKIITIGSICMVIGIVSLMLQIGNMISIWVSHSIQTGNQHQDEP--------------------IRNTNFLTE
A/Red-tailed_Hawk/BC/FAV-0053-31/2022_-_EPI_ISL_19154744_-_EPI3314000                   MNPNQKITTIGSICMVIGIVSLMLQIGNIISIWISHSIQTGNQYQPEPCNQSIITYE--------------------
A/common_raven/California/24_007417-001/2024_-_EPI_ISL_19094492_-_EPI3260120            MNPNQKITTIGSICMVIGIVSLMLQIGNIISIWVSHSIQTGNQYQPDPCNQSIITYENNTWV----------NFLAE
A/Est/Parnu/henharrier/TA21-26003/2021_-_EPI_ISL_19028670_-_EPI3173459                  MNPNQRIITTGSICMVIGIVSLMLQIGNIISIWVSHSIQTGNQYQPEP--------------------ISNTNFLAE
A/Est/Parnu/Seagull/TA21-13284-5/2021_-_EPI_ISL_19028669_-_EPI3173451                   MNPNQKITTIGSICMVIGIVSLMLQIGNIISIWVSHSIQTGNQYQPE--------------------PISNTNFLAE
```

Figure S4: Examples of different deletions appearing in the N1 stalk region of clade 2.3.4.4b H5N1 viruses. For the sequences highlighted in yellow there were more than one sample with the same deletion observed, while the other sequences have a single example.

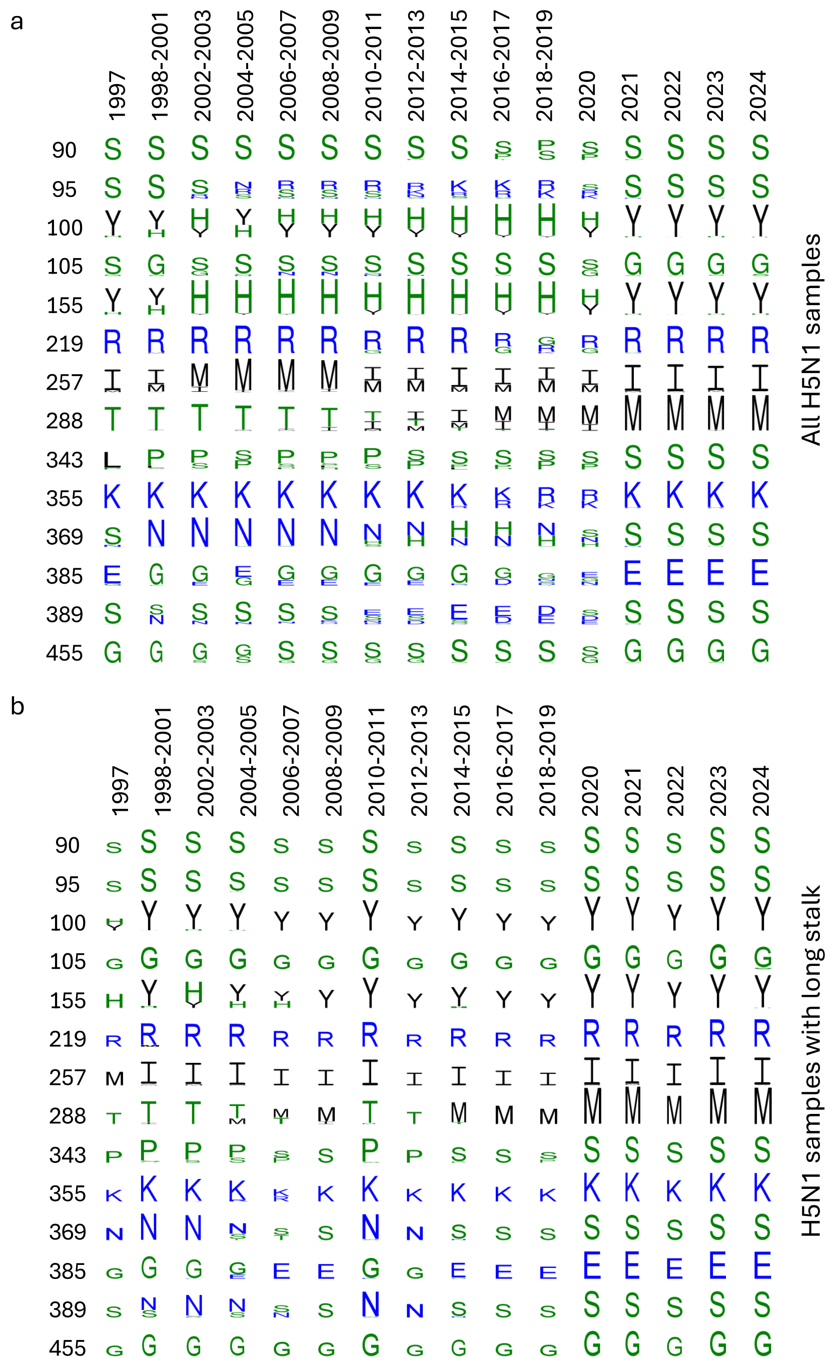

Figure S5. a) Amino acid positions that changed between 1998-2001 and 2018-2019, based on comparison of the two consensus sequences from these periods. For most positions the changes seemingly revert starting from 2020, to the variants observed in 1997 or 1998-2001. b) Comparison of the changes at the same positions only using the sequences that contain the NA with the long stalk from each dataset. Here, these positions mostly do not evolve from 1998-2001 to 2024. For both figures sequence logos were created using Weblogo 3. [12]. Numbering is N2 numbering, based on multiple sequence alignment with A/Perth/16/2009(H3N2) (PDB: 6BR5), using Clustal Ω.

```
A/Missouri/121/2024_-_EPI_ISL_19413343_-_EPI3556415      MNPNQKITTIGSICMVIGIVSLMLQIGNITSIWVSHSIQTGNQYQPEPCNQSIITYENNTWVNQTYINISNTNFLAEQAV
EMBOSS0001                                               MNPNQKITTIGSICMVIGIVSLMLQIGNIISIWVSHSIQTGNQYQPEPCNQSIITYENNTWVNQTYINISNTNFLAEQAV
                                                         ***************************** **************************************************
```

Figure S6: Comparison of the stalk region of the A/Missouri/121/2024 isolate to the consensus sequence of clade 2.3.4.4b previous to the collection of the Missouri sample. An I30T mutation appears, changing the NII site to an NIT site, adding another putative N-glycosylation site at the N28 position.

## References


[1] "NCBI Virus." Accessed: Aug. 08, 2024. [Online]. Available: https://www.ncbi.nlm.nih.gov/labs/virus/vssi/#/

[2] F. Sievers *et al.*, "Fast, scalable generation of high-quality protein multiple sequence alignments using Clustal Omega," *Mol Syst Biol*, vol. 7, p. 539, 2011, doi: 10.1038/MSB.2011.75.

[3] R. Gupta and S. Brunak, "Prediction of glycosylation across the human proteome and the correlation to protein function".

[4] F. Lemoine *et al.*, "NGPhylogeny.fr: new generation phylogenetic services for non-specialists," *Nucleic Acids Res*, vol. 47, no. W1, pp. W260–W265, Jul. 2019, doi: 10.1093/NAR/GKZ303.

[5] M. N. Price, P. S. Dehal, and A. P. Arkin, "FastTree: Computing Large Minimum Evolution Trees with Profiles instead of a Distance Matrix," *Mol Biol Evol*, vol. 26, no. 7, pp. 1641–1650, Jul. 2009, doi: 10.1093/MOLBEV/MSP077.

[6] M. N. Price, P. S. Dehal, and A. P. Arkin, "FastTree 2 – Approximately Maximum-Likelihood Trees for Large Alignments," *PLoS One*, vol. 5, no. 3, p. e9490, Mar. 2010, doi: 10.1371/JOURNAL.PONE.0009490.

[7] K. Katoh and D. M. Standley, "MAFFT Multiple Sequence Alignment Software Version 7: Improvements in Performance and Usability," *Mol Biol Evol*, vol. 30, no. 4, pp. 772–780, Apr. 2013, doi: 10.1093/MOLBEV/MST010.

[8] A. Criscuolo and S. Gribaldo, "BMGE (Block Mapping and Gathering with Entropy): A new software for selection of phylogenetic informative regions from multiple sequence alignments," *BMC Evol Biol*, vol. 10, no. 1, pp. 1–21, Jul. 2010, doi: 10.1186/1471-2148-10-210/FIGURES/9.

[9] "FastTree 2.1: Approximately-Maximum-Likelihood Trees for Large Alignments." Accessed: Sep. 17, 2024. [Online]. Available: http://www.microbesonline.org/fasttree/#Support

[10] I. Letunic and P. Bork, "Interactive Tree of Life (iTOL) v6: recent updates to the phylogenetic tree display and annotation tool," *Nucleic Acids Res*, vol. 52, no. W1, pp. W78–W82, Jul. 2024, doi: 10.1093/NAR/GKAE268.

[11] F. Madeira *et al.*, "The EMBL-EBI Job Dispatcher sequence analysis tools framework in 2024.," *Nucleic Acids Res*, vol. 52, no. W1, pp. W521–W525, Jul. 2024, doi: 10.1093/NAR/GKAE241.

[12] G. E. Crooks, G. Hon, J. M. Chandonia, and S. E. Brenner, "WebLogo: A Sequence Logo Generator," *Genome Res*, vol. 14, no. 6, p. 1188, Jun. 2004, doi: 10.1101/GR.849004.
